# Prognostic significance of thyroid or cricoid cartilage invasion in laryngeal or hypopharyngeal cancer treated with organ preserving strategies

**DOI:** 10.1186/1748-717X-7-219

**Published:** 2012-12-21

**Authors:** Marcus M Wagner, Joel K Curé, Jimmy J Caudell, Sharon A Spencer, Lisle M Nabell, William R Carroll, James A Bonner

**Affiliations:** 1 Departments of Radiation Oncology, University of Alabama at Birmingham, Hazelrig-Salter Radiation Oncology Center, 1700 6th Avenue South, Birmingham, AL, 35249-6832, USA; 2 Departments of Radiology, University of Alabama at Birmingham, Birmingham, AL, USA; 3 Departments of Medicine, and Otolaryngology, University of Alabama at Birmingham, Birmingham, AL, USA; 4 Departments of Otolaryngology, University of Alabama at Birmingham, Birmingham, AL, USA; 5 Department of Radiation Oncology, Moffitt Cancer Center, Tampa, FL, USA

**Keywords:** Thyroid, Cricoid, Cartilage, Invasion, Head and neck, Cancer, Local failure, Radiotherapy, Larynx, Hypopharynx

## Abstract

**Background:**

The utility of definitive radiotherapy (RT) for locoregionally advanced squamous cell carcinoma (SCC) of the larynx or hypopharynx in the setting of thyroid or cricoid cartilage invasion (TCCI) is controversial. A retrospective review of our experience was performed.

**Methods:**

Our institutional database of patients with SCC of the head and neck treated with radiotherapy (90% received concurrent systemic therapy) between 1995 and 2009 was queried. We identified 87 patients with T3-4 laryngeal or T4 hypopharyngeal cancer for whom initial head and neck imaging was available for review. Imaging of all patients was reviewed by a single radiologist specializing in neuroradiology. The presence and extent of TCCI was determined and used for stratification.

**Results:**

Median follow-up was 34 months. TCCI was found in 25 (29%) patients, eight limited to the inner cortex and another 17 involving both cortices. Local control (LC) was not significantly affected by TCCI limited to the inner cortex. However, TCCI involving both cortices was correlated with diminished LC at 2 years compared to the group of patients with no or minor invasion (55% vs. 81%, p=0.045). However, TCCI involving both cortices was not associated with significantly reduced rates of survival with a functional larynx, or overall survival (OS).

**Conclusions:**

Our results suggest that the rate of LC of T3-4 laryngeal or T4 hypopharyngeal SCC treated with definitive RT is not affected by TCCI of the inner cortex. Although decreased LC was significantly associated with TCCI involving both cortices, this factor did not appear to result in reduced rates of survival with a functional larynx or OS. Therefore, organ preservation may remain an option in these patients.

## Background

Patients with thyroid or cricoid cartilage invasion (TCCI) of the larynx were included in the landmark Veteran’s Administration (VA) Laryngeal Cancer Study Group trial which established the effectiveness of organ preservation for advanced laryngeal cancer using a regimen of induction cisplatin and 5-fluorouracil followed by radiotherapy (RT) [1]. Although there was a higher rate of salvage laryngectomy in patients with gross cartilage invasion, this difference was not significant. However, T4 tumors in general had a 59% risk of salvage laryngectomy, significantly higher than smaller tumors. The EORTC trial 24891 also tested a similar organ preservation scheme for locally advanced tumors of the pyriform sinus [2]. None of the four T4 patients randomized to induction chemotherapy achieved a complete response and were therefore diverted to definitive surgery.

Based on the findings of the VA and EORTC trials, the RTOG chose to exclude high volume T4 tumors in its largest laryngeal preservation trial, 91–11. High volume was defined as penetration through the cartilage or extending more than 1 cm into the base of the tongue [3]. In contrast, several European groups continued to include T4 lesions of the larynx and hypopharynx in organ preservation trials [4-7]. Given the uncertainty regarding feasibility of organ preservation in T4 laryngeal or hypopharyngeal cancers with TCCI, we retrospectively examined outcomes for this population at the University of Alabama at Birmingham.

## Methods

The University of Alabama at Birmingham Department of Radiation Oncology database of patients with squamous cell carcinoma (SCC) of the head and neck treated with definite radiotherapy between 1995 and 2009 was queried. Patients were included in this study whose AJCC staging criteria (version 6.0) incorporated TCCI (T3-4 laryngeal SCC or T4 hypopharyngeal SCC), were 18 years old or older, received definitive radiotherapy (RT), and had pretreatment imaging of the neck with either computed tomography (CT) or magnetic resonance imaging (MRI) that was available for review. Eighty-seven patients remained for analysis. These patients did not undergo surgery due to various reasons. Patients were reviewed at a multidisciplinary conference and their preferences as well as co-morbidities were considered. Of the 87 patients, 27 were treated on chemoradiotherapy protocols.

**Figure 1:**
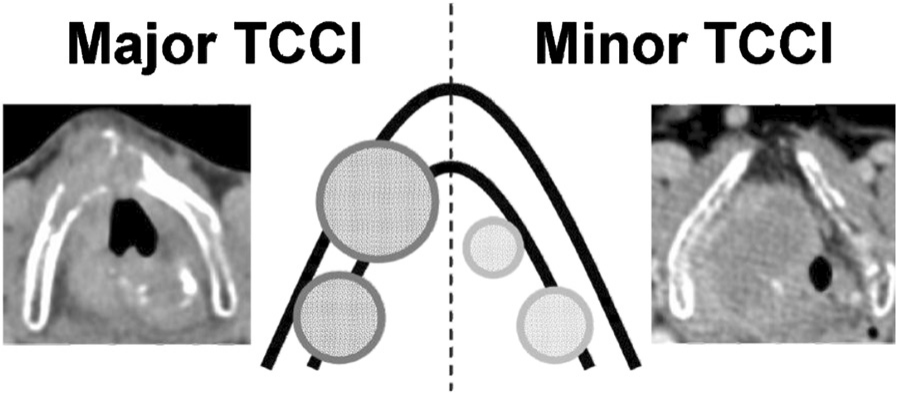
**Illustration and CT images of major (left) and minor (right) thyroid or cricoid cartilage invasion (TCCI).** The AJCC 7^th^ edition staging manual for laryngeal tumors classifies minor TCCI (i.e. invasion limited to the inner cortex) as T3 and major invasion (i.e. invasion into both inner and outer cortices) as T4. Both minor and major TCCI are staged as T4 for hypopharyngeal tumors.

A single radiologist (JKC), who specializes in neuroradiology, performed a detailed review of pre-treatment neck imaging to determine the presence or absence of TCCI. The extent of TCCI was further characterized as limited to the inner cortex (henceforth “minor TCCI”) or invading into both inner and outer cortices with or without extralaryngeal spread (henceforth “major TCCI”). Examples are shown in Figure 1. Patients judged to solely have sclerosis of the cartilage or extralaryngeal spread without cartilage invasion were not considered to have TCCI.

Treatment techniques for this patient population have been reported previously [8]. Briefly, all patients were immobilized with a thermoplastic head and neck mask and underwent CT simulation. Patients were planned using 3-dimensional treatment planning or intensity modulated radiation therapy (IMRT) with 6-megavolt photons. In this group of 87 patients, 16 patients had tracheostomies prior to radiation and this procedure was primarily performed for stabilization of the airway. Induction and concurrent chemotherapy consisted of a platinum agent with or without a taxane or fluorouracil. The anti-EGFR monoclonal antibody cetuximab was occasionally utilized with definitive radiation as previously described [8].

Patient characteristics were compared using the Chi-square test. All endpoints were calculated from the start of radiotherapy. Local control (LC), laryngectomy-free survival (LFS; defined as surviving patients without laryngectomy), laryngo-esophageal dysfunction-free survival (LEDFS; defined as surviving patients without local relapse, total or partial laryngectomy, or tracheotomy or feeding tube at two years or later as previously discribed [9]), and overall survival (OS) were calculated using the Kaplan-Meier method with the log rank test for comparisons. Statistical analyses were performed with SPSS version 19.0 (IBM Company, New York, USA).

## Results

Median follow-up for the 87 eligible patients was 34 months. Patient, tumor, and treatment characteristics are listed in Table 1. Median RT dose to the gross disease was 70.2 Gy (range 65.4 – 76.8 Gy). TCCI was found in 25 (29%) patients, eight with minor TCCI and 17 with major TCCI. The thyroid cartilage alone was involved in 18 patients, cricoid cartilage alone in two patients, and both in five patients. All but two cases of minor TCCI had extension of the primary disease beyond the larynx qualifying these tumors as T4.

**Table 1:** Patient, Tumor, and Treatment characteristics

**Characteristic**	**All Patients (n,%)**	**Any Invasion**
Total	87 (100)	25 (29)
Gender		
Male	68 (78)	19 (76)
Female	19 (22)	6 (24)
Age	Median = 57 years (range 33 – 81)	Median = 58 years (range 41 – 81)
Ethnicity		
Caucasian/Other	49 (56)	13 (52)
African-American	38 (44)	12 (48)
KPS score		
50 - 80	73 (84)	21 (84)
90 - 100	14 (16)	4 (16)
Primary site with T-stage		
**Larynx**	**78 (90)**	**21 (84)**
T3	44 (51)	2 (8)
T4a	30 (34)	18 (72)
T4b	4 (5)	1 (4)
**Hypopharynx**	**9 (10)**	**4 (16)**
T4	8 (9)	4(16)
T4b	1 (1)	0 (0)
N-stage		
N0	34 (39)	11 (44)
N1	11 (13)	6 (24)
N2	35 (40)	5 (20)
N3	7 (8)	3 (12)
Overall Stage		
III	24 (28)	1 (4)
IVA	53 (61)	20 (80)
IVB	10 (11)	4 (16)
Concurrent Therapy		
None	9 (10)	2 (8)
Cetuximab	6 (7)	1 (4)
Cytotoxic Chemotherapy	72 (83)	22 (88)
Neoadjuvant Chemotherapy		
No	75 (86)	21 (84)
Yes	12 (14) *	4 (16)
Fractionation of RT		
Conventional	39 (45)	9 (36)
Altered Fractionation	48 (55)	16 (64)
IMRT		
Yes	52 (60)	15 (60)
No	35 (40)	10 (40)
Local Recurrence		
Yes	17 (20)	7 (28)
No	70 (80)	18 (72)
Regional Recurrence		
Yes	5 (6)	1 (4)
Synchronous with LR	3 (60)	1(100)
Isolated	2 (40)	0 (0)
No	82 (94)	24 (96)
Distant Metastasis		
Yes	16 (18)	4 (16)
No	71 (82)	21 (84)

* 11 of 12 received concurrent chemoradiotherapy after induction and one received radiotherapy alone.

**Figure 2:**
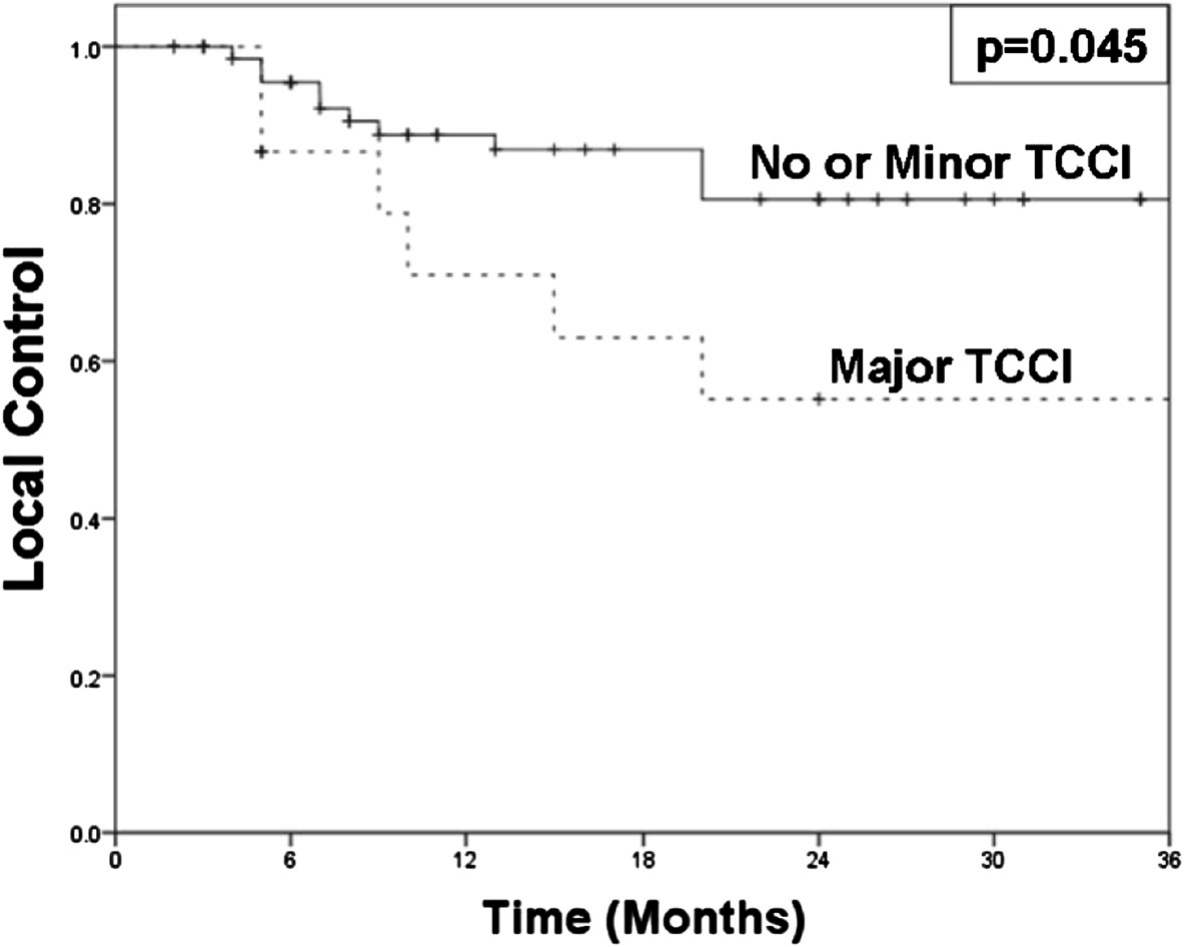
**Kaplan-Meier curves for local control comparing no or minor TCCI (solid) versus major TCCI (dashed).** Two-year local control for patients with no or minor TCCI was 81% compared to 55% with major TCCI (p=0.045).

Local failure occurred in 10 of 62 patients without TCCI, 1 of 8 patients with minor TCCI, and 6 of 17 patients with major TCCI. Two year LC was 80% (± 5.8% standard error) without TCCI, 86% (± 13.2% standard error) with minor TCCI, and 55% (± 13.8% standard error) with major TCCI (Table 2; p>0.05 for all comparisons). The patients with no TCCI or minor TCCI were found to have significantly higher 2 year LC compared to those with major TCCI (81% *vs*. 55%, p=0.045, Figure 2). Within the subset of patients with T4 disease (43 patients), major cartilage invasion (versus all other T4 cases) was not significantly associated with worse LC (55% *vs*. 78%, p=0.16). The six patients from the major TCCI group who had local failures did not have synchronous distant failures (though one had a synchronous regional failure) and were able to undergo salvage laryngectomy. Three of these patients were without evidence of recurrent disease more than two years after salvage surgery.

**Table 2:** Local Control and Overall Survival at 2 years

	**# of patients**	**Local control**	**P value**	**Overall Survival**	**P value**
All patients	87	76%		64%	
Any TCCI	25	65	0.15	64	0.86
No TCCI	62	80		65	
Minor TCCI	8	86	0.87	63	0.97
No TCCI	62	80		65	
Major TCCI	17	55	0.045	65	0.82
No or minor TCCI	70	81		64	

**Figure 3:**
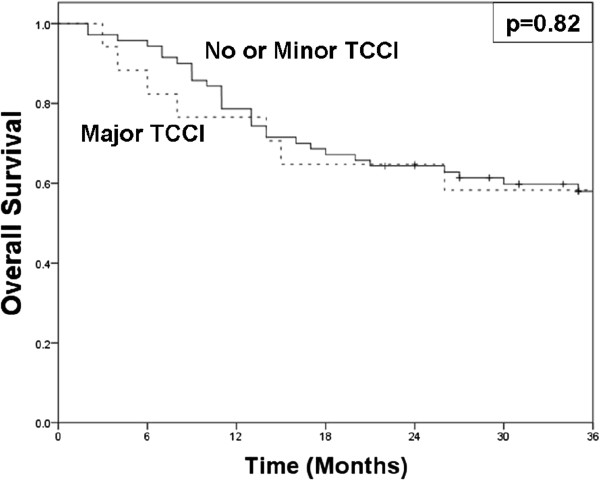
**Kaplan-Meier curves for overall survival comparing overall survival for no or minor TCCI (solid) versus major TCCI (dashed).** Two-year overall survival for patients with no or minor TCCI was 64% and 65% for major TCCI (p=0.82).

Although major TCCI was associated with decreased LC, there was no significant difference in rates of LFS at 2 years. The LFS for major TCCI was 41% compared to 58% for none/minor TCCI (p=0.31). In addition, the LEDFS at 2 years for major TCCI was 34% compared to 44% for none/minor TCCI (p = 0.38; Table 3). As shown in Figure 3, no difference in OS was found between major TCCI (65%) and no/minor TCCI (64%).

**Table 3:** Organ Preservation at 2 years

	**Laryngectomy-free survival**	**P value**	**Laryngo-esophageal dysfunction-free survival**	**P value**
All patients	55%		41%	
Any TCCI	44	0.28	35	0.45
No TCCI	60		44	
Minor	50	0.64	38	0.87
No TCCI	60		44	
Major TCCI	41	0.31	34	0.38
No or minor TCCI	58		44	

## Discussion

This study of patients with T3/T4 laryngeal cancer and T4 hypopharyngeal cancer revealed that major TCCI was a poor prognostic factor regarding LC and not LFS, LEDFS, or OS. Furthermore, within the subset of T4 tumors, major TCCI was not a significant predictor of worse LC. However, this latter analysis of T4 patients was limited due to small numbers of patients in this subgroup. Our results suggest that it may be reasonable to offer organ preservation with CRT for motivated patients with major TCCI. This proposal is based on the fact that patients who fail organ preservation have good salvage options and the lack of evidence that upfront surgery provides improved survival outcomes.

These results are supported by other reports of organ preservation strategies for advanced laryngeal and hypopharyngeal tumors. For example, Do et al. [10] retrospectively analyzed 101 patients with stage T4 head and neck cancers treated with definitive RT or resection, from several centers associated with the University of California Davis. Subset analysis of the CRT group found no differences in LC (50% and 56% at 5 years, respectively) or OS in 26 patients with bone and/or cartilage invasion compared to 42 patients with other T4 criteria. The same group later demonstrated no difference between bone or cartilage invasion [11]. Samant et al. [12] also reported no difference in outcomes based on bone versus cartilage invasion in 45 patients treated with intra-arterial cisplatin and concurrent RT (CRT). In contrast, Huang et al. [13] reported that patients with cricoid cartilage invasion had decreased 5 year OS (LC data not provided) based on multivariate analysis of 47 patients with hypopharyngeal cancer.

Various strategies are currently being tested to optimize the selection of patients for organ preservation. The University of Michigan [14] studied the combined results of two phase II trials in 36 patients with major TCCI by employing a chemoselection regimen. Treatment with CRT or surgery was determined by response to a single cycle of cisplatin and fluorouracil. After one cycle of chemotherapy, 81% of patients had a ≥50% response. These responders underwent CRT yielding 58% LFS and 78% OS at 3 years. Other groups have employed this same response-based approach and have found encouraging results in selective organ preservation based on restaging biopsies [15,16] or functional imaging [17] during CRT. Others have suggested that selection based on initial tumor volume [18] or pre-treatment laryngeal function [9] may be reasonable strategies. Further study of these innovative organ preserving strategies is warranted in the setting of locoregionally advanced head and neck cancers with cartilage invasion.

It is noteworthy that TCCI is commonly misdiagnosed by CT imaging. Beitler et al. [19] compared preoperative imaging with the results of subsequent surgical pathology following laryngectomy. Of the 107 clinically staged T4 laryngeal tumors, CT imaging correctly identified only 59% of cases with major cartilage invasion without extralaryngeal spread and 49% of cases with extralaryngeal spread [positive predictive values (PPV) 74% and 81%, respectively]. A similar study by Li et al. [20] reported only 60% identification of major cartilage invasion (100% if extralaryngeal spread was present) with CT imaging. Additionally, 47% of tumors identified as T4 by CT were downstaged to T3 after pathologic review. Determination of TCCI using MRI may have a higher detection rate for intracartilagenous invasion but is a costly and limited resource that is prone to false positive results [21]. We attempted to minimize our potential misinterpretation of TCCI by excluding findings of cartilage sclerosis and extralargyngeal spread without cartilage invasion and by systemically reanalyzing pre-treatment images.

Our results are subject to the inherent limitations of any retrospective review. First, only 17 patients had major cartilage invasion. Second, the occasional use of simulation scans, when diagnostic scans were not available, may have limited the detection rate of TCCI. Third, organ preservation therapy precludes pathologic review as the gold standard for determination of TCCI. With these noted caveats, the current analysis suggests that organ preservation strategies can be considered in patients with TCCI.

## Conclusions

Major TCCI were associated with decreased LC, but not LFS, LEDFS, or OS. These patients tend to fail locally (without distant spread) and are good candidates for salvage surgery. Without a clear survival advantage to initial surgery in this population, organ preservation may still be a reasonable treatment option.

## Competing interests

Drs. Wagner, Curé, Caudell, Spencer, Carroll, and Nabell: Actual or potential conflicts of interest do not exist.

Dr. Bonner: Occasional consultant / honoraria for Astra Zeneca, Bristol-Myers Squibb Company, Eli Lilly and Company, Genmab, ImClone Systems, Inc., Merck Serono, Oncolytics, Sanofi-Aventis and AVEO.

## Authors’ contributions

MMW, JJC and JAB are responsible for study conception and design. MMW, JKC, JJC and JAB participated in data collection and interpretation. MMW and JJC were responsible for statistical evaluation. All authors are responsible for manuscript writing. MMW, JJC and JAB are responsible for assembly of all figures and tables. All authors are in agreement with the content submitted herein. All authors read and approved the final manuscript.
